# The value of minilaparotomy for total hysterectomy for benign uterine disease: A comparative study with conventional Pfannenstiel and laparoscopic approaches

**DOI:** 10.1186/1755-7682-2-11

**Published:** 2009-04-22

**Authors:** Pedro Royo, Juan Luis Alcázar, Manuel García-Manero, Begoña Olartecoechea, Guillermo López-García

**Affiliations:** 1 Department of Obstetrics and Gynaecology, Clínica Universitaria de Navarra, Universidad de Navarra, Navarra, Spain

## Abstract

**Background:**

The aim of this paper is to review and compare the results obtained using the Pfannenstiel, laparoscopy and minilaparotomy approaches for total hysterectomy procedure in relation to benign uterine diseases.

**Methods:**

A retrospective data analysis was performed on 165 patients who underwent hysterectomy for benign uterine diseases at our centre during the period 2004 to 2006.

**Findings:**

The minilaparotomy procedure was the fastest procedure with a mean time of 73.4 minutes (range: 67.85 to 78.94 minutes, p < 0.001). Hospital stay was shortest for laparosopic procedure (mean time: 3.24 days, range: 2.86 to 3.61 days) (p < 0.001). The rate of intraoperative and postoperative complications were not statistical different among three procedures.

**Conclusion:**

The minilaparotomy procedure offers a minimally invasive option for total hysterectomy due to benign uterine disease.

## Background

Currently gynaecologists have different options for the surgical treatment of benign uterine diseases [1]. For the total hysterectomy operation, one of the most common procedures in gynaecological practice, the laparoscopic procedure has been widely accepted as a better alternative to Pfannenstiel laparotomy.

However, the minilaparotomy procedure (a transverse abdominal incision into the pubic hair no longer than 6 cm in length in which it is possible to place a circular elastic retractor that enables a better exposure of the pelvic field) is another possible option [2-4]

The aim of this study is to compare the results, in terms of morbidity, obtained following minilaparotomy, Pfannenstiel and laparoscopy approaches for total hysterectomy procedure.

## Methods

A retrospective data analysis was performed on 165 patients who underwent total hysterectomy for benign diseases only (fibroids, adenomyosis, dysfunctional uterine bleeding and endometrial hyperplasia) during the period 2004 to 2006. 81 patients (49.1%) had undergone the minilaparotomy procedure, 46 (27.9%) the Pfannenstiel laparotomy procedure and 38 (22%) the laparoscopic procedure. The choice of the procedure was based on surgeon's decision according to their preference.

The variables reviewed in the study were as follows:

1. Demographic data: age, height, and weight, body mass index (BMI), previous pregnancies, previous deliveries, and previous abdominal surgeries.

2. Uterine volume as estimated by ultrasound according to prolate ellipsoid formula (length × height × width × 0.5233, expressed in cm3) [5]

3. Type of uterine pathology.

4. Preoperative hemoglobin.

5. Intraoperative complications: Bladder, ureteral, bowel and vascular injuries and bleeding (measured as difference of preoperative and postoperative hemoglobin).

6. Postoperative complications: Wound infection, hematoma-seroma or dehiscence, urinary infection and pain (as measured by visual analog scale -VAS-).

7. Hospital stay and surgical time.

Three surgeons performed all surgical interventions at the Department of Obstetrics and Gynecology of the Clínica Universitaria de Navarra (Spain) with more than 10-year experience in all these techniques (only one surgeon for each surgical approach).

### Surgical technique for the Minilaparotomy procedure

Under general anaesthetic, the patient was placed in the lithotomy position.

Endovenous antibiotic prophylaxis administered was Cephazolin (2 g) or Clindamycin (600 mg/8 h) in penicillin-allergic patients. Subcutaneous Bemiparine (2500–3500 UI/24 h) was administered eight hours before surgery as thromboembolism prophylaxis.

**Figure 1:**
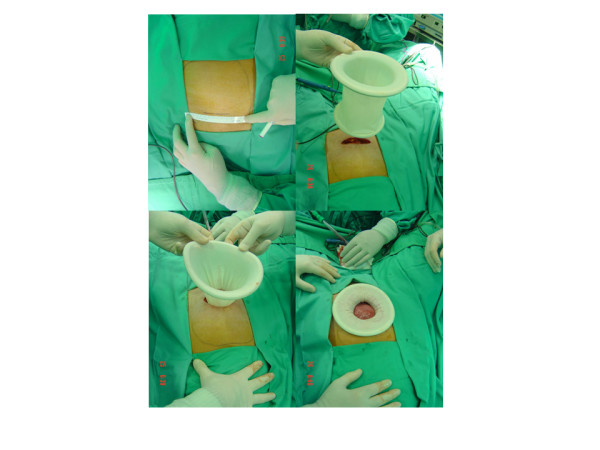
**Minilaparatomy access line measuring less than 6 cm**. Large uterus shape is also drawn above (top-left). Introduction of the self-retaining abdominal retractor (top-right). Introduction of the self-retaining abdominal retractor (down-left). Isolation of the uterus from other pelvic organs, atraumatically (down-right).

A Foley catheter was introduced within the bladder and a uterine manipulator was placed through the cervix for uterine mobilisation. Then a small transverse incision (3 to 6 cm in length) was made into the pubic hairline. A 6-cm incision creates a 28-cm^2 ^surgical area [3]. Once the pelvic cavity was reached, we placed a self-retaining abdominal retractor (Mobius, Apple Medical, Marlborough, MA) which is an elastic tubular device that isolates atraumatically the uterus from other pelvic organs (Figure 1).

**Figure 2:**
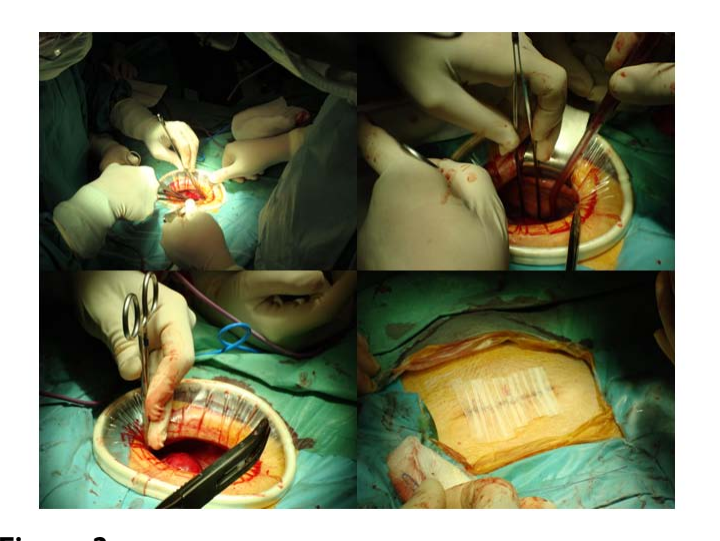
**Minilaparotomy hysterectomy being performed with conventional open-surgical instruments (top-left)**. Pelvic field exposed during surgery (top-right and down-left). Cosmetic outcomes after surgery (down-right).

The uterine ligaments and the vascular pedicles were ligated and cut with LigaSure (Valleylab, Boulder, CO) with the help of the second assistant who manipulates the uterine manipulator [4]. The first assistant presented the pelvic field using Sims and Deaver retractors when required. All hysterectomies were performed extra-fascially. If required, manual morcellation may help uterine abdominal extraction (Figure 2).

The post-operative analgesia protocol administered was endovenous Metamizol 2 gr/8 h and Acetaminophen 1 gr/6 h.

The laparoscopic and Pfannenstiel techniques were performed according to standard techniques [6,7]. These procedures were also performed using the LigaSure vessel-sealing system.

### Statistical analysis

As this is a retrospective study we did not calculate statistical power according to sample size.

All data were presented using the mean value with standard deviation and 95% confidence intervals. A p-value < 0.05 was considered as statistical significant.

The continuous variables were compared using ANOVA with Bonferroni post hoc test or Kruskall-Wallis tests. The Chi-square test was used for comparing categorical variables. All data analyses were performed using SPSS version 13.0 (SPSS, Inc., Chicago IL).

## Findings

165 hysterectomies were analysed: 81 (49.1%) were minilaparatomy procedures, 46 (27.9%) were Pfannenstiel procedures and 38 (23%) were laparoscopic procedures.

102 (62%) hysterectomies were performed to treat uterine myomatosis and 63 (38%) were performed due to the existence of other benign diseases. No statistical association was demonstrated between the surgical approaches and the type of uterine pathology (p 0.578) (Table 1).

**Table 1:** Indications according to surgical procedure.

	Minilaparotomy	Pfannenstiel	Laparoscopy	Total
Fibroids	56	27	19	102
Adenomyosis	4	2	2	8
Dysfunctional bleeding	18	15	15	48
Endometrial hyperplasia	3	2	2	7

Total	81	46	38	165

40 out of 165 (24.2%) patients had received previous abdominal surgery: 21 out of 81 (25.9%) of the minilaparotomies, 13 out of 46 (28.3%) of the Pfannenstiel laparotomies and 6 out of 38 (15.8%) of the laparoscopic procedures. No statistical association was demonstrated between the surgical approaches and the presence of previous surgeries (p 0.366).

The demographic and preoperative variables did not reveal significant association with regard to the surgical approaches (Table 2)

**Table 2:** Patients' demographic data according to surgical procedure.

	**Minilaparotomy**			**Pfannenstiel**			**Laparoscopy**		
	
	**Mean**	**Range**	**SD**	**Mean**	**Range**	**SD**	**Mean**	**Range**	**SD**
**Age (years)**	50	90-40	8	47	78-35	8	48	62-32	6
**Heigh (cm)**	160	172-142	6	162	187-152	6	162	186-150	7
**Weight (kg)**	64.02	100-47	10.10	64.78	94-35	14.75	64.53	96-40	1.33
**Body mass Index** **(kg/m2)**	25.05	44.44-16.46	4.40	24.84	37.73-14.38	5.70	24.61	37.50-15.03	4.73
**Pregnancies**	1	8-0	2	1	5-0	1	2	5-0	1
**Deliveries**	1	6-0	1	1	4-0	1	2	4-0	1
**Uterine volume (cm3)**	251.88	1204.79-5.27	235.24	292.38	1708.05-16.18	333.13	173.02	587.14-29.44	132.17
**Preoperative hemoglobin (gr/dl)**	12.8	15.8-8.4	1.4	12.6	15.5-8.5	1.6	12.4	16.1-8.6	1.6

N.S. for all comparisons

Mean surgical time for the minilaparotomy approach was significantly shorter (73.4 minutes, SD: 25.1, range: 67.8 to 78.9) as compared with the Pfannenstiel (101.9 minutes, SD: 32.2, range: 92.4 to 111.5. p < 0.001) and laparoscopy (159.3 minutes, SD: 58.1, range: 140.2 to 178.5. p <0.001). Laparoscopic hysterectomy resulted in the least number of days of hospitalization (mean: 3.2 days, SD: 1.1, range: 2.9 to 3.6) as compared with minilaparotomy (mean 3.9 days, SD: 1.0, range: 3.7 to 4.1. p = 0.023) and Pfannenstiel approach (mean 5.1 days, SD: 1.5, range: 4.6 to 5.5. p < 0.001)

Intraoperative bleeding and intraoperative complocations were similar for all three procedures analysed (Table 3)

**Table 3:** Intraoperative complications according to surgical procedure

	**Minilaparotomy**	**Pfannenstiel**	**Laparoscopy**
**None**	80	42	37
**Bladder injury**	0	2	0
**Ureteral injury**	0	0	1
**Bowel injury**	0	0	0
**Vascular injury**	0	0	0
**Active bleeding**	1	2	0
**Mean difference** **Pre and postoperative** **Hemoglobin (gr/dl)**	2.1 (SD: 1.0)	2.2 (SD: 1.4)	1.8 (sd: 1.2)

TOTAL	**81**	**46**	**38**

N.S. for all comparisons

Postoperative complications were also similar for all three procedures (Table 4).

**Table 4:** Postoperative complications according to surgical procedure.

	**Minilaparotomy**	**Pfannenstiel**	**Laparoscopy**
**None**	76	41	37
**Wall infection**	0	0	0
**Urinary infection**	0	0	1
**Hematoma-seroma**	2	4	0
**Suture dehiscence**	0	1	0
**Pain (VAS > 7)**	1	0	0
**Two or more**	2	0	0

TOTAL	**81**	**46**	**38**

N.S. for all comparisons

## Discussion

In the present study we have found that total hysterectomy by minilaparotomy is faster than Pfannenstiel and laparoscopic approach. These results confirm previous data from by Sharma [8] and Hoffman-Lynch [9]. We also found the minilaparotomy procedure to be faster than the laparoscopic and Pfannenstiel procedures. Although the shortest hospital stay was for the laparoscopic approach.

Regarding intraoperative and postoperative complications we did not find any statistical differences, so all three technique are similarly safe, in agreement with other previous studies [8-10].

One relevant question is that a small surgical area provides better cosmetic outcome [10]. In addition, the minilaparotomy procedure offers the potential to leave the abdominal surface free from scars (lending further cosmetic value to the process). Nevertheless, a reduced surgical field may also generate some difficulties in respect of pelvic access (large fibroids, presence of adherences) or upper abdominal cavity exploration (routine in the laparoscopic approach). This situation makes advisable the presence of a second surgical assistant using the uterine manipulator, as with regard to the laparoscopic process too. As a matter of fact, our surgeons did not find minilaparotomic approach more difficult than laparotomic or laparoscopic approaches.

The minilaparotomy technique may be considered an "atraumatic procedure" [8] because neither fixed abdominal retractors nor pneumoperitoneum [10] are used, which are both potential causes of postoperative pain (although this was not measured in our study).

Several studies have assesse the role of the minilaparotomy technique for the hysterectomy procedure, alone or in comparison with other abdominal or endoscopic approaches (Table 5) [1,3,8,9,11,12]. Most of these studies concluded that minilaparotomy may be an alternative to other approaches. However, it is difficult to compare the outcomes of these studies due to the heterogeneity of study designs, the different preoperative conditions and as well as the variables considered.

**Table 5:** Summary of published studies assessing minilaparotomy approach for total hysterectomy.

	**Hoffman&Lynch^9^**	**Panicci^1^**	**Sharma^8^**	**Fanfani^11^**	**Alcalde^3^**	**Muzii^12^**	**Present series**
**Study period**	1996–1997	1995–2001	1998–2002	1997–2003	2002–2005	2005	2004–2006
**Study design**	Retrospective	Prospective	Prospective non-randomized	Retrospective	Retrospective	Prospective randomized multicenter trial	Retrospective
							
**Comparison groups**	L, V, LAVH	L	L	-	-	L, LAVH	L, LH
**N** **Minilaparotomy/Total**	26/250	118/148	100/200	67	150	41/82	81/165
**Mean age** **(years)**	54 (28–84)	47 (38–75)	31.9 (32–50)	39.2 (26–52.4)	45 (45.3–47.7)	48(41–61)	50 (90–40)
**Body mass Index** **(kg/m2)**	???	25 (18–45)	???	23.6 (20.2–26.9)	24 (23.4–24.5)	???	25.05 (44.4–16.4)
**Minilap. size** **(cm)**	5–6	6–10	<6	4–9	<6	5–9	<6
**Self-retaning retractor**	No	Yes	No	Yes	Yes	No	Yes
**Only bening conditions**	No	Yes	Yes	Yes	No	Yes	Yes
**TAH +/- BSO**	Yes	Yes	Yes	Yes	No*	Yes	Yes
**Mean operative time (min)**	84 (45–125)	50 (34–88)	41 (30–90)	84.9 (53.1–116.7)	70 (63.1–70.7)	58 (45–75)	73.4 (67.8–78.9)
**Mean day of discharge**	3.4 (2–8)^†^	3 (2–5)	4.3 (3–8)	3.11 (2.3–3.8)	2.5 (2.4–2.6)	3 (1–5)^†^	3.8 (3.6–4.1)^†^
**Mean uterus weight** **(gr)**	123 (19–275)	285 (148–482)^†^	???	???	131.9 (113.9–149.8)	345 (180–600)^†^	251.8^‡^ (1204–5.27)^†^
**Postoperative bleeding** **(gr/dl)**	173 (75–500)^≈^	< 1.5	240^≈^	???	???	1.4 (0.2–2.1)	2.1 (5–0.1)

* 14.6% were STAH.

^† ^Expressed as median value.

^‡ ^Expressed as volume (cm^3^).

^≈ ^Expressed as ml.

The only prospective randomized multicenter trial performed so far by Muzzi et al concluded that LAVH was a better option than minilaparotomy because a shorter stay and lower morbidity [12].

## Conclusion

Minilaparotomy procedure may be considered a time saving technique for total hysterectomy for benign uterine pathology. It offers some of the advantages of a minimally invasive procedure (low morbidity, short hospital stay, good cosmetic results) and the benefits of open access (for example, shorter learning curve than laparoscopy).

## Abbreviations

TAH +/- BSO: Total abdominal hysterectomy +/- bilateral salpingoforectomy; STAH: Sub-total abdominal hysterectomy; L: Laparotomy; V: Vaginal approach; LAVH: Laparoscopic-assisted vaginal hysterectomy; LH: Laparoscopic hysterectomy; N.S: Not statistically significant.

## Competing interests

The authors declare that they have no competing interests.

## Authors' contributions

PR reviewed the literature, designed the study, collected all data, performed the statistical analysis and wrote the paper. JLA was responsible for the methodological and statistics corrections. MGM revised and corrected all areas in the text covering this field. BO revised and corrected all english lenguage areas of the paper. GLG coordinated all areas of research proccess.
